# DNA methylation changes in biomarker loci occur early in cancer progression

**DOI:** 10.12688/f1000research.21584.2

**Published:** 2020-02-14

**Authors:** Lukas Vrba, Bernard W. Futscher

**Affiliations:** 1 The University of Arizona Cancer Center, The University of Arizona, Tucson, Arizona, 85724, USA; 2 Department of Pharmacology & Toxicology, College of Pharmacy, The University of Arizona, Tucson, Arizona, 85724, USA

**Keywords:** DNA Methylation, Cancer Biomarker, Epigenetics

## Abstract

Tumor-specific DNA methylation can be used for cancer diagnostics and monitoring.  We have recently reported a set of DNA methylation biomarkers that can distinguish plasma samples from lung cancer patients versus healthy controls with high sensitivity and specificity.  Furthermore, the DNA methylation signal from the biomarker loci detected in plasma samples correlated with tumor size and decreased after surgical resection of lung tumors.  In order to determine the timing of DNA methylation of these loci during carcinogenesis and thus the potential of the biomarkers to detect early stages of the disease we analyzed the DNA methylation of the biomarker loci in five precancerous conditions using available data from the GEO database.  We found that the DNA methylation of the biomarker loci is gained early in carcinogenesis since most of the precancerous conditions already have biomarker loci hypermethylated.  Moreover, these DNA methylation biomarkers are able to distinguish between precancerous lesions with malignant potential and those that stay benign where data is available.  Taken together, the biomarkers have the potential to detect the earliest cancer stages; the only limitation to detection of cancer from plasma samples or other liquid biopsies is the timing when tumors start to shed enough DNA into body fluids.

## Introduction

Tumor cells have fundamentally different DNA methylation profile from normal cells of origin ^1– 4^. Some of these differences are tumor-specific, i.e. do not occur in any normal cell types, and thus could be used for tumor DNA identification. Since tumors shed DNA into bloodstream or other body fluids ^5– 7^, the detection of tumor-specific DNA methylation in these liquid biopsies could be utilized for non-invasive cancer diagnostics and monitoring ^8, 9^. This initiated a search for cancer-specific DNA methylation biomarker loci and analysis of these loci in plasma samples and other liquid biopsies ^10– 12^. We have previously described a large suite of cancer-specific DNA methylation biomarker loci discovered using TCGA and GEO data from over 10,000 tumor and normal samples ^13^. Recently, we developed qPCR amplicons specific for a subset of these biomarker loci designed to detect common carcinoma types and tested them on clinical cfDNA samples from healthy individuals and non-small cell lung cancer (NSCLC) patients. We demonstrated that these biomarkers can distinguish between healthy subjects and NSCLC patients with high sensitivity and specificity ^14^. Moreover, in blood samples from lung cancer patients the biomarker DNA methylation signal positively correlates with tumor size ^14^. The purpose of the current study was to find how early during carcinogenesis the biomarker loci gain DNA methylation in order to assess their potential as detectors of early stage cancer. To this end, we analyzed DNA methylation of the biomarker loci in publically available data from several precancerous conditions. We found that the biomarker loci gain DNA methylation early in carcinogenesis since they are methylated already in majority precancerous lesions analyzed; in addition, where the data are available, the markers can distinguish lesions with malignant potential from those that stay benign.

## Methods

The DNA methylation data from the Illumina HumanMethylation450 platform were downloaded from the GEO database (GEO accessions GSE60185, GSE66313, GSE53051, GSE58999, GSE48684, GSE77954, GSE72872, GSE81334, GSE108123 and GSE39279). These DNA methylation data are presented as beta values - numeric values in interval 0.0-1.0. For unmethylated CpGs the beta value approaches zero, for fully methylated CpGs beta approaches 1 and for CpGs methylated in a fraction of the sample 0<beta<1, e.g. a CpG methylated in 50% of the sample will have a beta value of approximately 0.5. All data were analyzed in the R programming environment, version 3.6.1 ^15^ as follows: The beta values were normalized as described ^13^. The normalized beta values for 10 biomarker CpGs ( Table 1, ^14^) were used in further analysis. Boxplots were created using the R function boxplot and the R library beeswarm,version 0.2.3. Since the beta values do not have normal distribution, nonparametric Wilcoxon rank-sum test was used to test differences between the groups. Multidimensional scaling (MDS) plots were constructed using the R function cmdscale on matrices of distances between samples and projected into two dimensions. The ability of the marker set to distinguish between progressive and regressive lung CIS was evaluated using receiver operating characteristic (ROC) analysis on the sums of the beta values from all 10 marker CpG Illumina probes ( Table 1). The ROC analysis and AUC calculations were performed using the R library pROC ^16^, version 1.15.3.

**Table 1.** List of 10 DNA methylation biomarker loci used in the study. The first column specifies Illumina CpG probe and the second column shows the genomic position of each biomarker CpG.

Illumina CpG.ID	CpG.position (hg19)
cg14416371	chr11:43602847-43602848
cg08189989	chr2:105459164-105459165
cg00100121	chr1:169396635-169396636
cg03306374	chr16:23847325-23847326
cg01419831	chr2:162283705-162283706
cg25875213	chr19:38183055-38183056
cg00339556	chr5:16180048-16180049
cg01893212	chr7:49813088-49813089
cg14732324	chr5:528621-528622
cg07302069	chr7:27196286-27196287

## Results and discussion

We have previously described a set of DNA methylation biomarker loci that are hypermethylated in 10 common carcinoma tumor types and we demonstrated that the level of DNA methylation of these loci can differentiate between plasma samples from lung cancer patients and healthy individuals. To determine the timing of the hypermethylation of these biomarker loci during human carcinogenesis and thus estimate potential of the markers to detect early disease stages we analyzed here the DNA methylation state of the biomarker loci in several premalignant conditions: breast ductal carcinoma *in situ* (DCIS), colorectal adenomas, Barrett’s esophagus (BE), pancreatic intraductal papillary mucinous neoplasms (IPMNs) and lung carcinoma *in situ* (CIS) using publically available Illumina HumanMethylation450 datasets from the GEO database.

**Figure 1.**
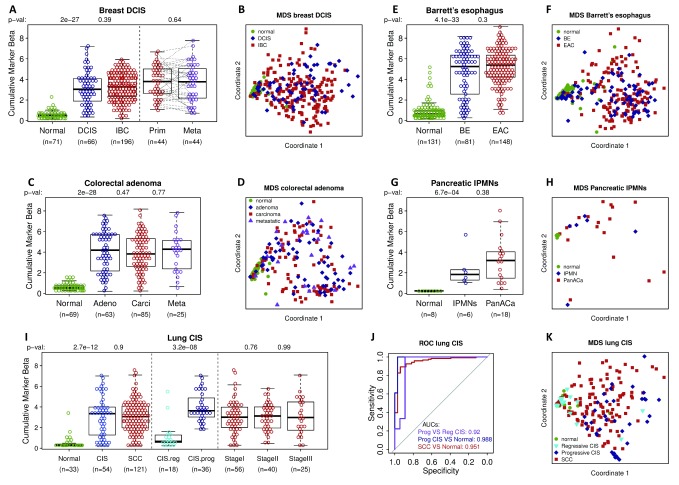
DNA methylation of the biomarker loci occurs early in carcinogenesis. The figure shows data from five primary carcinoma sites: breast ( **A**, **B**), colorectal ( **C**, **D**), esophagus ( **E**, **F**), pancreas ( **G**, **H**) and lung ( **I**, **J**, **K**). Boxplots ( **A**, **C**, **E**, **G**, **I**) show cumulative DNA methylation of the 10 biomarker loci in normal tissue samples, precancerous lesions, and tumor samples. The y-axes of plots represent the sums of beta values of the entire set of 10 biomarker CpG Illumina probes. The p-values (Wilcoxon rank-sum test) depicted at the top indicate significance of differences between neighboring groups of samples. The breast boxplots ( **A**) in addition show DNA methylation of the biomarker set in 44 pairs of primary breast tumors and lymph node metastases. The lung boxplots ( **I**) in addition present the precancerous lesion (carcinoma *in situ* (CIS)) cohort split into progressive and regressive sub cohorts and lung tumor (squamous cell carcinoma (SCC)) cohort split into stages I-III. The multidimensional scaling (MDS) plots ( **B**, **D**, **F**, **H**, **K**) show multidimensional scaling of pairwise distances derived from beta values of 10 biomarker CpG probes of the same sample cohorts as in the boxplots. The receiver operating characteristic (ROC) plot ( **J**) shows that the cumulative DNA methylation of the 10 biomarkers can differentiate progressive lung CIS samples not only from normal lung tissue samples (blue curve), but also from regressive lung CIS samples (purple curve). DCIS, ductal CIS; IPMN, intraductal papillary mucinous neoplasm.

Ductal carcinoma *in situ* is a precursor of invasive breast carcinoma (IBC). We analyzed DNA methylation of the biomarker loci in normal breast tissue samples, DCIS and IBC from three GEO datasets: GSE60185 ^17^, GSE66313 ^18^, GSE53051 ^19^. The results ( Figure 1A) show that the biomarker loci are methylated already in DCIS at about the same level as in IBC. The multidimensional scaling (MDS) plot ( Figure 1B) shows DCIS samples scattered among IBC samples, indicating comparable levels of DNA methylation of individual markers, while most of the normal samples form a small cluster on a side of the plot. Furthermore, there is no significant increase in the marker methylation during the progression to metastatic disease, as illustrated by data from a cohort (GSE58999 ^20^) of 44 pairs of primary breast tumors and lymph node metastases ( Figure 1A).

Colorectal adenomas are the precursor neoplasms to colorectal cancer. We analyzed biomarker loci in normal colorectal tissue, colorectal adenomas, colorectal carcinomas and metastatic colorectal tumors from three GEO datasets: GSE48684 ^21^, GSE77954 ^22^, GSE53051 ^19^. Similar to DCIS, biomarker loci are already hypermethylated in colorectal adenomas with no further increase in methylation during the progression into invasive colorectal carcinomas or metastatic colorectal cancer ( Figure 1C) and again colorectal adenomas on MDS plot are scattered among colorectal carcinomas ( Figure 1D).

Barrett’s esophagus is a precancerous precursor of esophageal adenocarcinoma (EAC). We analyzed normal esophagus together with BE and EAC samples from two GEO datasets: GSE72872 ^23^, GSE81334 ^24^. Again, similar to DCIS or colorectal adenomas, biomarker loci are hypermethylated already in BE ( Figure 1E, F). Similar situation was observed also in pancreatic intraductal papillary mucinous neoplasms (IPMNs), precursor lesions of pancreatic adenocarcinomas, where only one small dataset (GSE53051 ^19^) was available ( Figure 1G, H).

Finally, we analyzed lung CIS. Lung CIS is a pre-invasive precursor lesion of lung squamous cell carcinoma (SCC), one of the two non-small cell lung cancers that we previously used to demonstrate the capability of the biomarkers to distinguish between clinical plasma samples from cancer patients and healthy subjects. We analyzed DNA methylation of the biomarker loci in lung CIS together with lung SCC and normal lung tissue samples from GEO datasets GSE108123 ^25^, GSE39279 ^26^. The advantage of the original lung CIS study (GSE108123 ^25^) is that the prospective follow-up information is available for CIS samples and thus the samples could be classified as either progressive (those later progressed into invasive cancer) or regressive (these later regressed to normal epithelium or low-grade disease). Our analysis revealed, similar to the other pre-invasive lesions, that the biomarker loci have increased DNA methylation already at the lung CIS stage ( Figure 1I). More importantly, when we analyzed progressive and regressive lung CIS samples separately ( Figure 1I), we found that the biomarker set is able to distinguish between the two types of premalignant lesions with high sensitivity and specificity (AUC = 0.92, Figure 1J). The majority of the regressive lung CIS samples on the MDS plots cluster close to normal lung controls while all progressive lung CIS samples are scattered among lung SCC samples ( Figure 1K). Even when lung SCC samples are sub-grouped into the individual cancer progression stages (I-III) there is no increase in DNA methylation with the stage ( Figure 1I). Together, these results show that the gain of DNA methylation of the biomarker loci is an early epigenetic event during human carcinogenesis.

The data presented here show that DNA methylation of the biomarker loci is fundamentally changed early during the malignant progression since it is already observed in precancerous lesions. The data from lung CIS further show that the DNA methylation level of the biomarkers can differentiate between potentially malignant and benign CIS. Together, these findings indicate that the biomarkers are capable, from the qualitative point of view, to detect cancer at its earliest stages. However, the detection of cancer-specific DNA methylation in blood or other body fluids is quantitative in nature and depends on the tumor size and its propensity to shed DNA into bloodstream; e.g., our previous report ^14^ shows that the DNA methylation signal from this biomarker set in cfDNA samples depends on the NSCLC tumor size. Later disease stages are thus relatively easy to detect since larger tumors of later cancer stages shed a large amount of DNA into bloodstream resulting in high DNA methylation signal. In order to detect the early cancer stages as well, sensitive detection techniques and especially sample processing leading to minimal background DNA methylation signal will be profound to distinguish cancer from healthy samples. This report shows that the DNA methylation change of the biomarker loci is already present to its full extent in the earliest cancer stages. Thus, the combination of the sensitive detection and the timing of the release of enough tumor DNA into blood or other body fluids are the factors that will set the limit of the biomarkers to detect cancer early.

The early occurrence of the increased DNA methylation at biomarker loci may raise a question about the suitability of the biomarkers to screen patients with premalignant conditions for cancer. This will likely depend on the type of samples used for the screening. Premalignant lesions, due to their limited size and localized nature are unlikely to compromise screening in blood or other body fluids that is, as discussed above, quantitative in nature. Premalignant lesions are less likely to shed as much DNA as tumors to increase the level of methylated marker copies in blood derived cfDNA to the same threshold as the malignant disease. Therefore, once there is large enough increase of the biomarker signal in blood it might be indicative of malignant progression. The proper DNA methylation signal threshold will have to be determined by testing known clinical samples. On the other hand, the early hypermethylation of the marker loci would have to be considered if premalignant tissue samples were tested directly, e.g. small biopsies from Barrett’s esophagus patients; in this case it would be qualitative assay, high level of DNA methylation would be detected and samples falsely classified as malignant. However, collection of small tissue biopsies is more invasive than collection of blood or other liquid biopsies for which this biomarker set was originally designed ^14^. In addition, in the case of lung CIS the biomarkers were able to distinguish biopsies from premalignant lesions with malignant potential from those that stay benign, so the elevated signal from the biomarkers would be indicative when to treat the disease even in the premalignant stage. Overall, the fact that the biomarker loci are hypermethylated already in precancerous lesions have to be taken into account when using the biomarkers for screening patients with premalignant conditions, but it does not diminish the utility of the biomarkers for noninvasive cancer diagnostics.

In conclusion, the biomarker loci have the potential to detect malignant disease at its earliest stage and the only limitation to the use of the biomarkers to detect cancer from liquid biopsies is the timing when the tumors start to release enough DNA into bloodstream.

## Data availability

### Source data

Illumina HumanMethylation450 DNA methylation data used in the presented study can be downloaded from the Gene Expression Omnibus database ( https://www.ncbi.nlm.nih.gov/geo/; Accession numbers: GSE60185, GSE66313, GSE53051, GSE58999, GSE48684, GSE77954, GSE72872, GSE81334, GSE108123 and GSE39279).
