# Supplementary material for: DNA methylation changes in biomarker loci occur early in cancer progression
Source: F1000Res. 2020 Feb 14;8:2106. Originally published 2019 Dec 16. [Version 2] doi: 10.12688/f1000research.21584.2 (PMC6993824; doi:10.12688/f1000research.21584.2)
Supplement: Peer review history

# Peer review: Reviewer response for version 1

**DOI:** 10.5256/f1000research.23787.r57862

**Published:** 2020-01-24

**Authors:** Judd C. Rice

**Affiliations:** 1 Department of Biochemistry and Molecular Medicine, Keck School of Medicine, University of Southern California, Los Angeles, CA, USA

In this brief report by Vrba and Futscher, the authors sought to leverage their previous discovery of 10 cancer-associated DNA methylation biomarker loci to investigate the clinical potential of these biomarkers in assessing the progression of carcinogenesis. The authors analyzed several publicly available datasets that included normal tissue, precancerous lesions and tumor samples to generate *in silico* models of breast, colorectal, esophageal, pancreatic and lung cancer progression. The rigor of the study design is high as all datasets utilized the same technology (Illumina HumanMethylation450) with appropriate sample size of each group for analysis, except for the pancreatic samples. Although the data presented support the authors’ conclusions, at least by the eyeball test, the inclusion and description of detailed statistical analyses would increase confidence in the conclusions. The overall results indicate that aberrant methylation of the biomarker loci is an early epigenetic event of carcinogenesis, regardless of cancer type. This is an important finding that further supports the clinical potential of DNA methylation biomarkers in early cancer detection.

I confirm that I have read this submission and believe that I have an appropriate level of expertise to confirm that it is of an acceptable scientific standard.

# Response

**Published:** 2020-02-11

**Authors:** Lukas Vrba

**Affiliations:** The University of Arizona Cancer Center, USA

Dear Dr. Rice,

Thank you for the evaluation of our study. According to your suggestion and suggestion from the other reviewer we added the p-values to Figure 1. The results now show that all the differences that made the “eyeball test” are also highly statistically significant, including in the pancreatic samples where the n is rather low but the control cohort is consistently unmethylated. We thank you for this suggestion, since the amended version of our manuscript will have higher impact among the readers.

# Peer review: Reviewer response for version 1

**DOI:** 10.5256/f1000research.23787.r57865

**Published:** 2020-01-08

**Authors:** Keith D. Robertson

**Affiliations:** 1 Department of Molecular Pharmacology and Experimental Therapeutics, Mayo Clinic, Rochester, MN, USA

In this manuscript, Vrba and Futscher examined the DNA methylation status of candidate loci in pre-malignant stages of a variety of cancers (e.g. breast, pancreas, and esophagus). Ultimately, the authors observed that DNA hypermethylation events previously identified in full-blown cancer were already hypermethylated in the precancerous tissues. One particularly interesting outcome was that progressive lung carcinoma *in situ* demonstrated elevated methylation levels, while regressive CIS more closely resembled normal lung tissue, suggesting that these CpGs may actually be predictive of progression and/or play a role in carcinogenesis.

 This is a straight-forward *in silico* analysis study, and seems worthy of indexing since the methylation biomarker field is moving rapidly and showing significant promise.

 This reviewer has two concerns, however, that should be addressed:

1. While the methylation trends are clearly qualitatively visible, statistical significance is not given for any of the comparisons.
2. Due to pre-malignant lesions showing similar patterns as the corresponding cancer, does that reduce the efficacy of these biomarkers as the target screening population malignancy is indistinguishable from the cancer? For example, healthy individuals are not routinely screened for esophageal carcinoma, but those with Barrett's esophagus are.

I confirm that I have read this submission and believe that I have an appropriate level of expertise to confirm that it is of an acceptable scientific standard.

# Response

**Published:** 2020-02-11

**Authors:** Lukas Vrba

**Affiliations:** The University of Arizona Cancer Center, USA

Dear Dr. Robertson,

Thank you for the prompt and generally positive review of our article. We have postponed our response until all reviews were available and a new version of the article was submitted.

As to the first point raised, the other reviewer had the same suggestion and therefore we added p-values for comparisons of individual sample cohorts. All differences between normal tissues and respective premalignant lesions are highly statistically significant, while there are no significant differences in DNA methylation of the biomarker loci during further malignant progression.

We especially appreciate your second point. The presence of the increased DNA methylation at marker loci may indeed raise doubts about how well the biomarkers may serve for cancer screening of individuals with premalignant conditions. We think that it depends on the type of the samples used for the screening (liquid vs tissue biopsies) and we have added the whole new paragraph discussing this topic. We thank you for raising this important point since it had to be discussed to make the study complete.
